# Curcumin nanoparticles: the topical antimycotic suspension treating oral candidiasis

**DOI:** 10.1007/s10266-022-00742-4

**Published:** 2022-09-13

**Authors:** Souzy Kamal Anwar, Sarah Nasser Abd Elmonaem, Eglal Moussa, Aliaa Gamaleldin Aboulela, Marwa Morsy Essawy

**Affiliations:** 1 Oral Medicine, Periodontology, Oral Diagnosis, and Oral Radiology Department, Faculty of Dentistry, Alexandria University, Alexandria, 21521 Egypt; 2 Microbiology Department, Medical Research Institute, Alexandria University, Alexandria, 21521 Egypt; 3 Oral Pathology Department, Faculty of Dentistry, Alexandria University, Alexandria, 21521 Egypt; 4 Center of Excellence for Research in Regenerative Medicine and Applications (CERRMA), Faculty of Medicine, Alexandria University, Alexandria, 21521 Egypt

**Keywords:** Nanotechnology, Nanocurcumin, Nystatin, Oral candidiasis

## Abstract

Phytotherapeutics is widely used nowadays as an alternative to the current antifungal drugs to reduce their side effects. Curcumin, with its wide therapeutic array as antioxidant and anti-inflammatory agent, is one of the natural compounds that ha..s an antifungal effect, especially when being used at nanoscale to increase its bioavailability. Our research aimed to evaluate clinically and microbiologically the effect of using topical nanocurcumin suspension to treat oral candidiasis. After 4 days from induction of oral candidiasis (baseline), we randomly divided 39 female BALB/c mice into three groups of 13 animals; nanocurcumin, nystatin, and sham groups. All animals in nanocurcumin and nystatin groups received topical treatment twice daily for 10 days. Then, we performed clinical and microbiological evaluations at baseline, day 5, and day 10. By the end of treatment, our results revealed that nanocurcumin promoted a significant reduction in the number of candida colonies. There was no statistically significant difference neither clinically nor microbiologically between nanocurcumin and nystatin groups. In conclusion, nanocurcumin has a good antifungal effect as nystatin, however, its therapeutic efficacy takes a longer time to appear than nystatin. The enhanced bioavailability of curcumin at the nanoscale qualifies this nano-herb as a promising alternative therapy for oral candidiasis, evading nystatin-associated morbidity.

## Introduction

Oral candidiasis is one of the most virulent opportunistic infections caused by an overgrowth of Candida yeasts, mainly *Candida albicans*, and is associated with counted morbidity [1, 2]. Of the current antifungals, nystatin is one of the extensively used topical and systemic antimycotics that interfere with the biosynthesis or the integrity of the cell wall of Candida [3]. However, prolonged use of nystatin has several drawbacks, including toxicity and the development of drug resistance, with an increased incidence of recurrence rate [4–6].

Studies have searched for novel antifungal drugs against these unmanageable fungal infections [7, 8]. The need for effective compounds of low toxicity leads to the entrance of natural products into treatment strategies of oral candidiasis. Curcumin, a perennial herb, is considered a promising natural antifungal. Curcuma longa has proven antioxidant, anti-inflammatory, antiproliferative, anti-invasive, and antiangiogenic activities [9–12].

As an antimycotic agent, curcumin can inhibit fungal growth through different mechanisms; by the generation of reactive oxygen species; interfering with the ergosterol biosynthesis pathway; altering the development of hyphae; and modulating multidrug efflux pumps [13].

Curcumin, however, is of poor water solubility, limiting its oral bioavailability and absorption with enhancing its metabolism and systemic elimination [14, 15]. Alternatively, curcumin is readily soluble in organic solvents. However, the reported toxicity of the organic solvents limits their biological uses [16]. Previously, our team documented the toxicity of acetone-dissolved curcumin in reducing the life span of rats in our verification path for candidal management. Furthermore, dryness of the tongue and oral cavity was observed only in survived animals treated with topical curcumin, in addition weight loss and reported anorexia. Meanwhile, water-soluble nanocurcumin revealed superior biocompatibility with a prolonged life span of the animals [17].

Nanotechnology has resolved the problems encountered with water-insoluble medications. Synthesis of hydrophobic drugs at a nanoscale enhances their solubility and improves their bioavailability. Moreover, the nanosized drugs enter the cells easily with a high affinity to target specific cytosolic sites such as proteins, nucleic acids, and other small molecules [18, 19].

Hence, our study aimed to evaluate the therapeutic effect of topical application of nanocurcumin suspension for the treatment of oral candidiasis in a murine model, using conventional treatment with nystatin suspension as a positive control, and an untreated group of mice as a negative control.

## Materials and methods

### Study design, sample size estimation and randomization

This comparative experimental study design complied with the ARRIVE guidelines and the guidelines laid down by the National Institute of Health (NIH) in the USA regarding the care and use of animals for experimental procedures. This study also followed the ethical guidelines for reporting in vivo experiments in animal research by the Faculty of Dentistry, Alexandria University (IRB No. 00010556-IORG 0,008,839).

For this study, we selected 42 female BALB/c mice, about 6 to 8 weeks old, weighing approximately 20–25 g. The animals were adapted to the standard laboratory conditions of temperature, humidity, and light/dark cycle (12 h/12 h) and were given a standard diet during the study period.

The sample size was estimated based on assuming confidence level = 95% and study power = 80%. Dovigo et al. [20] have reported mean ± SD log^10^ of *C. albicans* after nanocurcumin = 0.26 ± 0. 59 colony-forming units per millilitre (CFU/mL) while Sakima et al. [21] have reported mean ± SD after nystatin = 3.37 ± 1.85, respectively. The minimum sample size was calculated to be 13 mice per group, which increased to 14 mice per group to make up for possible sample attrition.

Sample size was based on Rosner’s method [22] calculated by G*power 3.0.10 software [23].

The animals were divided into three groups of 13 mice each because three died before treatment. The animals were randomly assigned using a computer-generated list of random numbers [24] to one of three groups: nanocurcumin, nystatin (as positive control), and sham (as negative control) groups.

### Synthesis and characterization of curcumin nanoparticles

We synthesized nanocurcumin by solvent-antisolvent precipitation method following our optimized protocol. The curcumin powder (Alpha Chemika, Mumbai, India) was dissolved in acetone (10 mg/mL). One mL of the prepared solution was added to 15 mL deionized water (ultra-purified from Millipore Milli-Q system “resistivity $\sim$ 80 MΩ cm”) containing 0.5% *w*/*v* polyvinyl pyrrolidone (PVP; Sigma-Aldrich, St. Luis, MO, USA) in a drop-wise manner under stirring at 500 rpm for 1 min [25].

The synthesized curcumin nanoparticles were characterized by ultraviolet–visible (UV–Vis) spectrophotometry (Nanodrop, DeNovix, DS-11 FX + , US) to measure the absorption spectra between 200 and 800 nm. The mean particles’ size and the surface charge were measured by dynamic light scattering analysis using Zeta-seizer (Nano ZS, Malvern Instruments, Worcestershire, UK). Transmission electron microscope (TEM; JOEL, JSM-6360LA, JAPAN) examination of the nanoparticles was conducted to assess the shape, size, and distribution of the particles [25, 26].

Nanocurcumin used at a minimum inhibitory concentration of 64 µg/mL against *C. albicans* ATCC90028 strain [27].

### Induction of Candida infection

We induced oral candidiasis in mice following Takakura et al. [28] and Mima et al. [29]. To exclude oral candidiasis before any procedure, we swabbed mice tongues to ensure a preliminary negative culture of *Candida* species. All animals were immunosuppressed with two subcutaneous injections of prednisolone (Egyptian Pharmaceutical Industries Co., Egypt) at a dose of 100 mg/kg body weight 1 day before and 3 days after infection with *Candida* species. Tetracycline hydrochloride (Chemical Industries Development, Egypt) in drinking water at a concentration of 0.83 mg/mL was given to the animals, starting one day before inoculation until the end of the study, to disturb the normal oral bacterial flora in the mice and facilitate candida colonization.

The reference strain *C. albicans* ATCC 90,028 (Virginia, USA) was used in the investigation. It was sub-cultured on Sabouraud Dextrose Agar (SDA) culture medium (Oxoid, Thermofisher inc., UK) supplemented with chloramphenicol (100 mg/litre) and incubated aerobically at 37 °C for 48 h. Then the colonies were suspended in sterile saline and the suspension was adjusted to a final concentration of 10^7^ CFU/mL.

To induce oral infection, the animals were sedated with chlorpromazine hydrochloride 0.1 mL (2 mg/mL) and then small cotton pads were soaked in a *C. albicans* cell suspension, and the dorsal surface of the tongues of the animals was swabbed for 1 min. Within 3 days after inoculation, 3 mice died.

After 4 days from induction of infection, a cotton swab was rolled twice over the tongues of all animals and used to inoculate SDA plates supplemented with chloramphenicol. After 48 h of aerobic incubation at 37 °C, the detected growth of *Candida* confirmed the successful induction of oral candidiasis before the beginning of the treatment.

### Treatment of the animals

The animals were randomly divided into three groups, each of 13 mice, after microbiological confirmation of the presence of oral *Candida* infection (baseline). The nanocurcumin treated group received nanocurcumin at the concentration of 64 µg/mL topically twice daily [27]; the positive control group received nystatin 100,000 U/mL topically twice daily [30]; the sham group as a negative control group did not receive any treatment. All animals in nanocurcumin and nystatin groups received the topical treatment using an oral dropper and oral brush. The mice were lightly sedated with 0.1 mL chlorpromazine hydrochloride and immobilized during dosing in a supine position till the suspension was topically applied all over their tongues. The time of application was standardized to one minute.

The treatment was administered for 10 continuous days, after which the experiment was terminated by euthanizing all animals with an intramuscular injection of a lethal dose of ketamine (> 30 mg/kg), according to the guidelines of the Faculty of Medicine, Alexandria University. After the animals were euthanized, their tongues were dissected and fixed in 10% buffered neutral formalin for histopathological examination.

### Clinical macroscopic evaluation

Clinical macroscopic evaluation was done at baseline after confirmation of candida induction, day 5, and day 10 of treatment by inspection and taking photographs to evaluate the progression of lesions on the tongue of the animals. Each photograph was rated using scores according to the classification proposed by Takakura et al. [28]. This classification divided the lesion by scoring from 0 to 4 based on the extent and severity of whitish, curd-like patches on the tongue surface as follows: 0, normal; 1, white patches in less than 20%; 2, white patches in less than 90% but more than 21%; 3, white patches in more than 91%; 4, thick white patches like pseudomembranes in more than 91%.

### Microbiological analysis

Samples were collected at baseline, day 5, and day 10 of treatment by rolling sterile cotton swabs over the dorsal surface of the tongue of all animals. The end of the cotton swab was then cut off, placed in a tube containing 1 mL of sterile saline, and vortexed for 1 min to resuspend the yeast cell, then 10 µL of this yeast suspension was used to inoculate in SDA plates supplemented with chloramphenicol for 48 h. The *Candida* colonies were counted, and the number of viable cells was determined to calculate the number of CFU/mL.

### Histopathological examination

For histopathological assessment of the treatment, the tongue specimens were processed for Periodic acid Schiff (PAS) stain to detect the candida hyphae/spores and to assess the inflammatory score, taking normal tongue specimens before candida induction as a negative control. Five microscopical fields were examined by microscope (Olympus BX41) at × 400 magnification connected with a digital camera (Olympus DP20).

The extent of infection and inflammation was scored and evaluated based on Zhang et al. [31] with a modification as follows, (absent scoring 0) for the absence of hyphae/spores and inflammation; (mild scoring 1) for the presence of hyphae/spores in the upper 1/3 layer of the tongue mucosa and 1–3 micro-abscesses in the epithelial layer; (moderate scoring 2) for the presence of hyphae/spores in the upper 2/3 layer of the tongue mucosa with 4–6 micro-abscesses in the epithelial layer and some neutrophils in the submucosa; (severe scoring 3) for hyphae/spores present in the whole mucosal layer of the tongue mucosa with > 6 micro-abscesses or large abscess formation in the epithelial layer and numerous neutrophils in the submucosa.

### Statistical analysis

Data were fed to the computer and analyzed using IBM SPSS software package version 20.0. (Armonk, NY: IBM Corp). The Shapiro–Wilk test was used to verify the normality of the distribution of variables; Comparisons between groups for categorical variables were assessed using the Chi-square test (Fisher or Monte Carlo). Kruskal Wallis test was used to compare different groups for abnormally distributed quantitative variables and followed by Post Hoc test (Dunn’s for multiple comparisons test) for pairwise comparison. Friedman test was used for abnormally distributed quantitative variables, to compare between more than 2 periods or stages and Post Hoc Test (Dunn’s) for pairwise comparisons. Significance of the obtained results was judged at the 5% level.

## Results

### Synthesis of stable curcumin nanoparticles with high yielding capacity

**Fig. 1:**
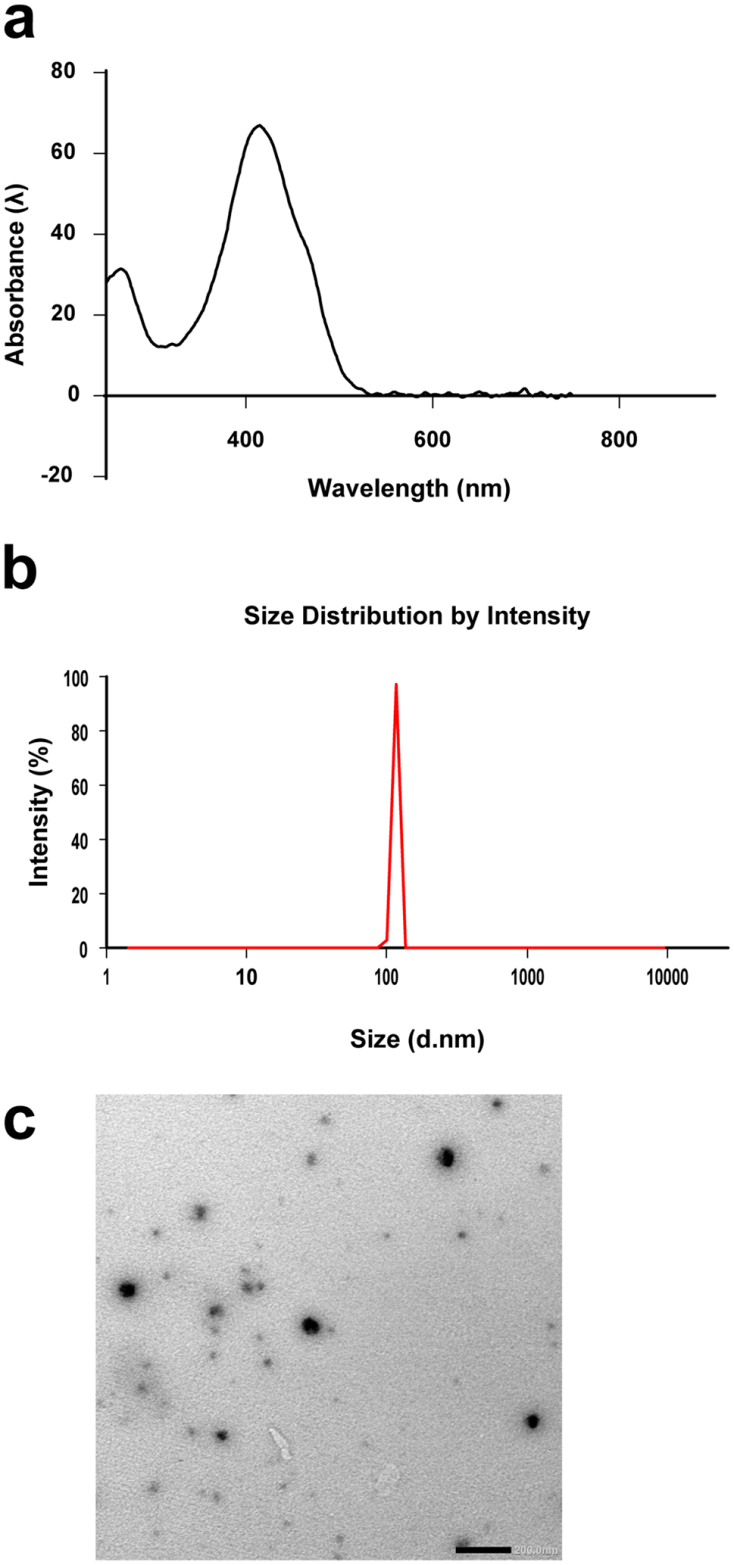
Photograph showing characterization of nanocurcumin: **a**, **b** the UV–Vis spectrophotometer characterization results for average particles’ size and Zeta potential. **c** curcumin nano particles examination with transmission electron microscope

Following our optimized solvent-antisolvent precipitation method, the synthesized nanocurcumin was highly stable with a narrow size range. The UV–Vis spectrophotometer revealed the characteristic absorbance peak of curcumin nanoparticles at 419 nm. Meanwhile, the dynamic light scattering analysis showed average particles’ size of 122.0 ± 2.704 nm, with a surface charge of − 20.2 ± 4.48 mV (Fig. 1a and b).

By transmission electron microscope examination, the curcumin nanoparticles exhibited regular spherical shapes with a particle size of 87.61 ± 2.34 nm (Fig. 1c).

### Remission of clinical candida scoring nearly to normal with topical application of nanocurcumin (Fig. 2)

**Fig. 2:**
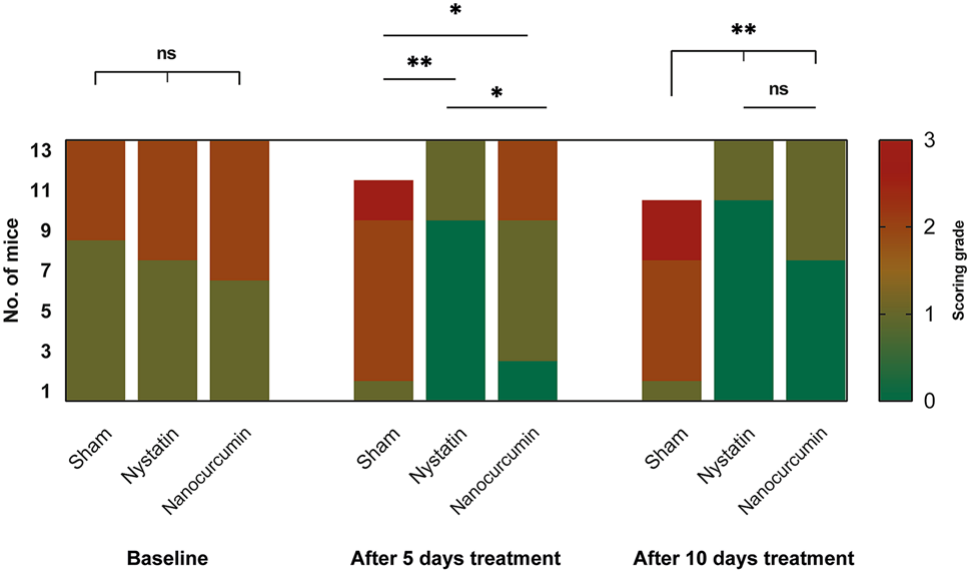
A bar chart showing the comparison between the three studied groups according to clinical scores. Significance is denoted by * for *p* < 0.05 and ** for *p* < 0.001, while ns denotes non-statistically significant

The immunosuppression of mice rendered them susceptible to induction of oral candidiasis. Four days after oral inoculation of *Candida albicans* ATCC 90028, oral candidiasis was microbiologically confirmed by a positive pure culture of *Candida albicans* from oral swabs obtained from all groups of mice under study.

Clinically, successful candidal induction in all animals was manifested as white patches/pseudomembranes with/out red denuded areas on the tongue dorsum. Upon examination at day 4 (baseline) after the inoculation, half of the animals in each group showed localized white candidal lesions in less than 20% of the tongue scoring 1. Meanwhile, the other half revealed extensive red denuded areas alternating with white pseudomembranes occupying 75% of the dorsal surface of the tongue with a score of 2.

After 5 days of treatment, a comparison of the clinical scores using the Kruskal–Wallis test showed that there was a statistically significant difference among the three study groups (*p* < 0.001).

Mice in the positive control (nystatin-treated) cohort showed a statistically significant improvement in the clinical scores compared with the negative control (sham) group (*p* < 0.001), with 69% showing a clinical score of 0 and 31% showing a score of 1.

On the other hand, after 5 days of treatment, clinical improvement of oral lesions was also observed in mice treated with nanocurcumin, although it was of a lower degree in comparison with the nystatin-treated group. The majority of mice (53.8%) showed a clinical score of 1, with 15.4% reverting to a clinical score of zero, and 30.8% retaining the clinical score of 2.

The improvement of clinical lesions after 5 days of treatment was statistically significantly higher with nystatin topical application in comparison with nanocurcumin (*p* = 0.011), using Dunn-Bonferroni test. However, both treatment modalities were statistically significantly effective in achieving improvement in clinical scoring compared with the negative control group; *p* = 0.022 for the nanocurcumin group, and *p* < 0.001 for the nystatin group.

After 10 days of treatment, there was no statistically significant difference between the clinical scores of the nystatin and the nanocurcumin-treated group (*p* = 0.416). On the other hand, a highly statistically significant difference was observed between the nystatin and nanocurcumin-treated groups, compared with the negative control group (*p *< 0.001).

Mice treated with nanocurcumin showed marked clinical improvement at day 10 of treatment, with nearly 53.8% reverting to a clinical score of zero, indicating complete clinical remission, and 46.2% showing a clinical score 1. On the other hand, no marked change in clinical scoring was observed in mice treated with topical nystatin, as only one mouse from the nystatin group turned from score 1 to score 0, while the other three animals maintained score 1.

Although nystatin showed a faster antifungal effect than nanocurcumin, there was no statistically significant difference between their therapeutic effects clinically by the end of the 10 days treatment period.

### The sturdy antimicrobial effect of nanocurcumin

**Fig. 3:**
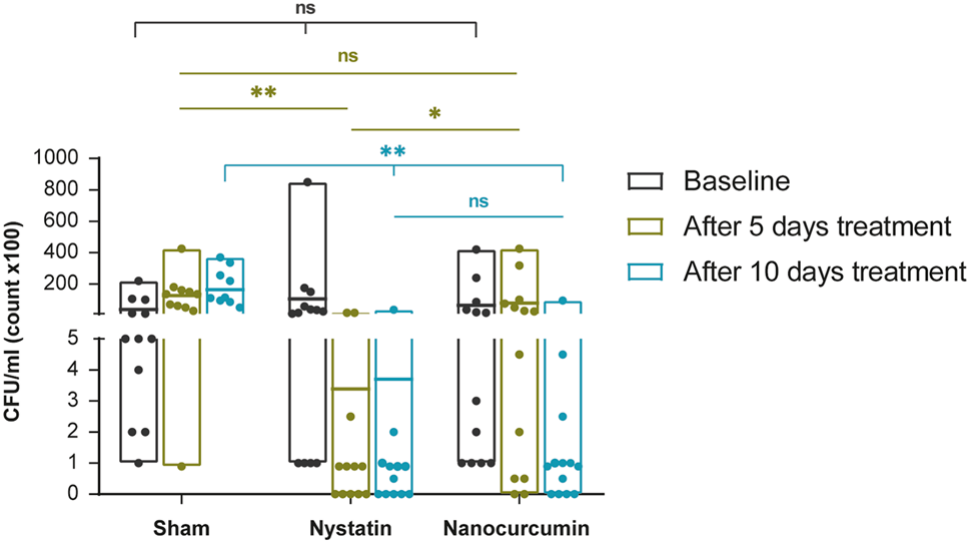
A representative graph showing a comparison between the three studied groups according to colony counts (CFU/ML) in each period. Significance is denoted by * for *p* < 0.05 and ** for *p* < 0.001, while ns denotes non-statistically significant

Before treatment commencement, the quantitative culture revealed a similar number of colonies among the three study groups (*p* = 0.723), ensuring the steadiness of *Candida* induction and that the 3 groups are comparable to each other **(**Fig. 3; Table 1).

**Table 1:** Comparison between the three studied groups according to colony counts (CFU/ML) in each period

Colony counts (CFU/ML)	Nanocurcumin	Nystatin	Control	*p*	*p*_1_	*p*_2_	*p*_3_
After induction	(*n* = 13)	(*n* = 13)	(*n* = 13)				
Median (IQR)	600 (5950)	2500 (11,550)	500 (5300)	0.723	> 0.05	> 0.05	> 0.05
After 5 days	(*n* = 13)	(*n* = 13)	(*n* = 11)				
Median (IQR)	2500 (8725)	50 (450)	13500 (11000)	< 0.001^a^	0.027^a^	0.067	< 0.001^a^
After 10 days	(*n* = 13)	(*n* = 12)	(*n* = 10)				
Median (IQR)	50 (175)	50 (175)	11250 (19800)	< 0.001^a^	0.688	< 0.001^a^	< 0.001^a^
p_0_	0.011^a^	0.015^a^	0.007^a^				
After induc. vs. 5 day	0.769	0.025^a^	0.014^a^				
After indu. vs. 10 day	0.008^a^	0.019^a^	0.004^a^				
5 days vs. 10 day	0.019^a^	0.919	0.655				

Pairwise comparison bet. each 2 groups was done using Post Hoc Test (Dunn’s for multiple comparisons test)

^a^Statistically significant at *p* ≤ 0.05

*p*: *p* value for Kruskal–Wallis test for comparing between the three studied groups

*p*_0_: *p* value for Friedman test for comparing between the three studied periods in each group

*p*_1_: *p* value for Chi-square test for comparing between nanocurcumin and nystatin

*p*_2_: *p* value for Chi-square test for comparing between nanocurcumin and control

*p*_3_: *p* value for Chi square test for comparing between nystatin and control

After five days of treatment, nystatin markedly reduced the colony count (CFU/mL) in comparison with the negative control (*p* < 0.001; Fig. 3; Table 1). However, nystatin did not eradicate *Candida* shortly, as indicated by the absence of the negative cultures after five days. Moreover, there was no statistically significant difference in the number of negative cultures among the three study groups (*p* = 0.073; Table 2).

**Table 2:** Comparison between the three studied groups according to number of negative cultures

No. of negative cultures	Nanocurcumin	Nystatin	Control	^MC^*p*	*p*_1_	^FE^*p*_2_	^FE^*p*_3_
5 days	2/13 (15.4%)	5/13 (38.5%)	0/11 (0%)	0.073	> 0.05	> 0.05	> 0.05
10 days	4/13 (30.8%)	6/13 (46.2%)	0/10 (0%)	0.038^a^	0.420	0.104	0.019^a^

*MC* Monte Carlo, *FE* Fisher Exact

^a^Statistically significant at *p* ≤ 0.05

*p*: *p* value for Chi-square test for comparing between the three studied groups

*p*_1_: *p* value for Chi-square test for comparing between nanocurcumin and nystatin

*p*_2_: *p* value for Chi-square test for comparing between nanocurcumin and control

*p*_3_: *p* value for Chi-square test for comparing between nystatin and control

Concerning the nanocurcumin group, despite the observed increase in *Candida* colonies count after five days of treatment, this increase was not statistically significant (*p* = 0.769). The nanocurcumin revealed its prompt antimycotic efficacy over time, where the number of colonies decreased significantly from day 5 to day 10 of treatment (The median count decreased from 2500 to 50; *p* = 0.019; Table 1).

Nanocurcumin was as efficient as nystatin in combating oral candidiasis with time, where both therapeutic agents reduced the colony count similarly at the end of the 10-day treatment period (*p* = 0.688; Fig. 3; Table 1). Moreover, the percentage of negative cultures in the nanocurcumin group showed a two-fold increase from 15.4 to 30.8% compared with the nystatin group, which only increased from 38.5 to 46.2% (Table 2).

### The antimycotic efficacy of nanocurcumin traced histologically

**Fig. 4:**
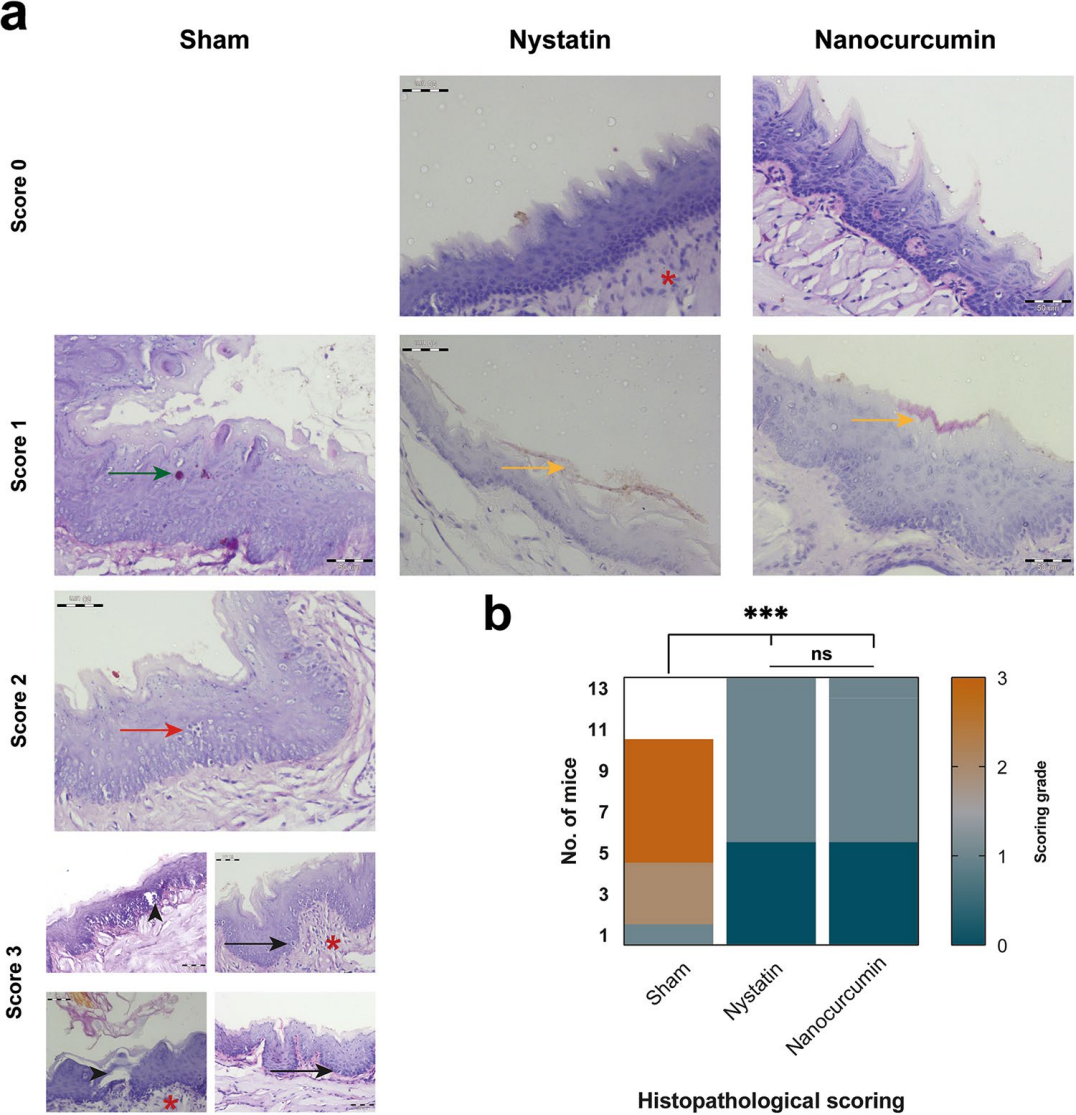
Histopathological assessment and scoring of the three different treatment modalities. **a** Photomicrographs of PAS-stained tongue specimens at the end of treatment period (× 400 with a scale bar of 50 µm). The untreated negative controlled tongue mucosae range in their scores from mild to severe. In mild score 1, the upper third of mucosa reveals candida (green arrow). While mild neutrophil infiltrations are noted in score 2 resulting in the formation of micro abscess (red arrow), which become more evident throughout the mucosal surface with Munro’s macro abscesses (arrowheads) in score 3. Red asterisks denote the neutrophils infiltration in the underlying connective tissue, while black arrows mark the mitotic activity in untreated candida infected tongue mucosa. Nystatin and nanocurcumin topical treatment restore the normal tongue architecture, with a scoring range from absent (0) to mild (1) revealing candida hyphae restricted to the keratinized epithelial layer (yellow arrows). **b** A heatmap of histopathological candida scoring system after 10-days treatment protocols showing the comparable significant drops in the scores to almost absent 0 grade in both nystatin and nanocurcumin treated groups (ns; *p* > 0.05), compared with the significant high histological candida count scoring mainly 2 and 3 (***; *p* < 0.0001)

At the end of the treatment period, the untreated group revealed significant severe histopathological changes, with a median score of 3 regarding the extent of spores throughout the tongue mucosa and the degree of inflammatory responses. Untreated candida resulted in the formation of many micro/macro-Munro’s abscesses, with the presence of candida spores deep to the basal one-third of the epithelium. Interestingly, Candida-infected epithelium revealed a degree of dysplastic changes ranging from moderate to severe epithelial dysplasia with evident mitotic activity. Upon topical treatment with nanocurcumin suspension, the histological picture significantly returned to almost normal tongue mucosa with bared counted hyphae in the upper one-third of the epithelial surface restricted to the cornified layer (*p* < 0.0001). The success of restoring the tongue mucosa histologically, with a median score of 0 and absence of inflammatory response to nanocurcumin treatment was comparable to the positively controlled samples treated with nystatin, (*p* > 0.05; Fig. 4).

## Discussion

Curcumin is a good antifungal compound, with intrinsic anti-inflammatory and antioxidant properties, however, its use is very limited due to its poor water-solubility [15, 32]. Curcumin nanoformulations have been introduced to solve this problem [33–36]. This study prepared nanocurcumin by a solvent-antisolvent precipitation method, which is a suitable technique for synthesizing highly soluble nanocurcumin [25]. The average particle size of nanocurcumin was 122.0 ± 2.704 nm to enhance absorption.

In our study, female mice were used to induce oral candidiasis. They provided a useful tool to evaluate the topical antifungal effect of the nanocurcumin clinically and microbiologically.

Clinical evaluation of the present study was done by inspection and taking photographs. Each photograph was rated using scores according to the classification proposed by Takakura et al. [28]. This classification depends based on the extent and severity of whitish, curd-like patches on the tongue surface. In our study, all animals infected with *C. albicans* showed red or/and white patches/pseudomembranes on the tongue dorsum with a score 1 or 2 of the lesions upon the first examination after induction of oral candidiasis. After the treatment period, all animals in the nanocurcumin and nystatin groups showed better clinical scoring with scores of 0 and 1 of the lesions compared to the scores before treatment. However, most of the animals of the negative control group maintained score 2 and three of them showed score 3 by the end of the treatment.

Mice treated with topical nystatin showed the best scoring after treatment with 10 animals turning to score 0 and only 3 animals maintaining score 1. In our study, the antifungal nystatin was used in a positive control group as a comparison parameter because it is considered the most commonly used topical antifungal to treat oral candidiasis [37].

In another study that used topical nystatin once daily, all animals showed score 1 after 5 days of treatment [38]. On the other hand, in our study 69% of the animals showed score 0 after the same treatment period. This better clinical outcome after the same duration of treatment could be attributed to the application of nystatin twice daily in the present study.

Many studies evaluated the therapeutic effect of curcumin. It has a good antifungal effect against many fungal species, including Candida in vitro, but it has poor pharmacokinetics in vivo [10, 39, 40]. Thus, in our study, we chose to evaluate the topical antifungal effect of nanocurcumin in vivo and compare it with conventional topical treatment with nystatin suspension.

For the nanocurcumin group, clinical improvement occurred after 10 days of treatment, with a score 0 in seven animals (53.8%) and a score 1 in 6 animals (46.2%). Topical treatment with nanocurcumin needed more time than nystatin to achieve clinical improvement of the tongue lesions.

When comparing the clinical scores between the nystatin and the nanocurcumin group, there was a statistically significant difference in favor of nystatin, only after 5 days of treatment. However, by the end of the treatment, there was no significant difference between them. Nanocurcumin has as good antifungal effect as nystatin, but it needs more time to treat oral candidiasis clinically.

The use of nanocurcumin for alleviation of oral inflammatory symptoms was also evaluated. The authors reported that nanocurcumin can be successfully used as an alternative to corticosteroids for the treatment of pain and reduction of lesion size of oral lichen planus, whenever corticosteroids are contraindicated [41].

In this study, the nystatin-treated group showed a high percentage of negative cultures (6 animals, 46%) at the end of the 10 days treatment period, based on a twice-per-day dose regimen. This percentage is close to the result of Bassiri-Jahromi et al. [42]; who reported that 40% of the cultures were negative after 10 days of treatment with nystatin, although they used a once-per-day dosing regimen. This indicates that nystatin has a robust antifungal effect that supports its validity as a positive control for comparative evaluation of the antifungal effect of novel antifungal agents such as nanocurcumin.

Comparing the colony counts across time between day 5 and day 10 in nanocurcumin treated groups, the significant reduction in candida counts points out the promising antimycotic effect of nanocurcumin, qualifying its use in the management of resistant oral candidiasis. However, nanocurcumin needed longer time to express its antifungal activity in comparison with other studies, which have reported better antifungal efficacy of nanocurcumin over a short time. This may be attributed to the difference between our low dose of nanocurcumin used topically to reduce the probability of its ingestion versus the higher doses that have been administrated systemically [27, 39].

Regarding the nystatin group, despite its short-term antifungal effect representing the significant reduction in the number of colonies after 5 days of treatment, the difference in the number of colonies between both time intervals (5 and 10 days) of treatment was not significant. In another study, nystatin has promoted a significant reduction in yeast count after 5 days of treatment. However, its sustainable effect has been questionable after 7 days of treatment cessation, where the number of colonies has increased significantly, suggesting infection recurrence [38]. This indicates that the significant reduction in the colony counts after a short time of nystatin treatment is not an indication of total curation and the evaluation must be extended for a longer time, with weighing the adverse effect of prolonged nystatin treatment.

Histologically, the topical application of nanocurcumin succeeded to restore the tongue mucosa, with a median score of 0 and absence of inflammatory response treatment in comparable to the positively controlled samples treated with nystatin. Similarly, curcumin-silk fibroin nanoparticles (CM-SF NPs) have protected the lung mucosa of the mice from damage by *C. albicans* infection [27]. Additionally, dendrosomal nanocurcumin has decreased significantly the gross pathological lesions in the organs of the mice in relation to the systemic dose used in the treatment [39].

Regardless of the varieties in curcumin nanoplatforms used in different studies, nanocurcumin has shown a robust antimycotic effect on a wide range of doses. This might be attributed to the difference in fungal species tested and the way of nanocurcumin administration. Combating the same species of *Candida* used in our study, Xue et al. [27] have reported the superior in vitro antifungal effect of CM-SF NPs over the curcumin in the bulk form, with the reduction of the minimum inhibitory concentration (MIC) to the half. However, systemic administration of CM-SF in vivo has raised the MIC, with preservation of its significant therapeutic efficacy in comparison with curcumin.

Another dendrosomal form of nanocurcumin has been evaluated for its in vitro and in vivo antifungal effect against different *Candida* species, using a higher concentration than that evaluated in the current study. The authors have reported that nanocurcumin has at least two-fold higher antifungal effect than curcumin in vitro. Moreover, intraperitoneal administration of nanocurcumin at the concentration of 40 mg/kg for the treatment of systemic disseminated candidiasis has decreased the fungal load significantly in the organs evaluated microbiologically [39].

In another in vitro study, the MIC of nanosized curcumin against two fungal strains (*P. notatum* and *A. niger*) other than candida species were 200 and 350 µg/mL, respectively [43].

Hence, our topical nanocurcumin has a promising antifungal effect, but further studies are recommended to modulate its concentration and the time needed for effective treatment without recurrence.

## Conclusion

As aforementioned, we can conclude that the topical application of nanocurcumin needs more time than nystatin to reduce the number of colonies significantly. Still, it has a good antifungal effect microbiologically as nystatin. Therefore, suspension of curcumin nanoparticles can be considered a new treatment modality for oral candidiasis to avoid nystatin-associated toxicity. Further studies are highly recommended to modulate its concentration and determine the optimum duration of treatment. Moreover, for the outstanding inherited optical properties of the curcumin at the nanoscale, ranging from its auto-luminescence used in cellular tracking of drug delivery to its photoactivation counted for enhancing its efficacy, the therapeutic profile of nanocurcumin requires further verifications.

## Data Availability

All data included in this current study are available from the corresponding author upon request.
